# Supplementary material for: The efficacy of DPP IV inhibitors as adjunct therapy for patients with auto-immune Diabetes: A systematic review and meta-analysis
Source: PLoS One. 2025 Sep 30;20(9):e0332191. doi: 10.1371/journal.pone.0332191 (PMC12483218; doi:10.1371/journal.pone.0332191)

# Appendix

## Search strategy:

### Pubmed Search query

("type 1 diabetes mellitus" OR LADA OR "juvenile diabetes" OR "type 1b diabetes" OR "idiopathic diabetes" OR "immune mediated diabetes" OR "ketosis prone diabetes mellitus" OR "autoimmune diabetes" OR IDDM OR JOD OR "latent autoimmune diabetes of adults") AND (Januvia OR sitagliptin OR saxagliptin OR Onglyza OR linagliptin OR Trajenta OR alogliptin OR Vipidia OR vildagliptin OR Galvus OR Eucreas OR Janumet OR Kombiglyze OR Vipdomet OR Jentadueto OR "dpp4 inhibitors" OR "dipeptidyl peptidase 4 inhibitors"), the same search strategy was used in the other databases

### Scopus search query

(("type 1 diabetes mellitus") OR (LADA) OR ("juvenile diabetes") OR ("type 1b diabetes") OR ("idiopathic diabetes") OR ("immune mediated diabetes") OR ("ketosis prone diabetes mellitus") OR ("autoimmune diabetes") OR (IDDM) OR (JOD) OR ("latent autoimmune diabetes of adults")) AND ((januvia) OR (sitagliptin) OR (saxagliptin) OR (onglyza) OR (linagliptin) OR (trajenta) OR (alogliptin) OR (vipidia) OR (vildagliptin) OR (galvus) OR (eucreas) OR (janumet) OR (kombiglyze) OR (vipdomet) OR (jentadueto) OR ("dpp4 inhibitors") OR ("dipeptidyl peptidase 4 inhibitors"))

Medline search query

| # | Query |
| --- | --- |
| 1 | (type 1 diabetes mellitus or LADA or juvenile diabetes or type 1b diabetes or idiopathic diabetes or immune mediated diabetes or ketosis prone diabetes mellitus or autoimmune diabetes or IDDM or JOD or latent autoimmune diabetes of adults).mp. [mp=title, book title, abstract, original title, name of substance word, subject heading word, floating sub-heading word, keyword heading word, organism supplementary concept word, protocol supplementary concept word, rare disease supplementary concept word, unique identifier, synonyms, population supplementary concept word, anatomy supplementary concept word] |
| 2 | exp *Diabetes Mellitus, Type 1/ |
| 3 | 1 or 2 |
| 4 | (januvia or sitagliptin or saxagliptin or onglyza or linagliptin or trajenta or alogliptin or vipidia or vildagliptin or galvus or eucreas or janumet or kombiglyze or vipdomet or jentadueto or dpp4 inhibitors or dipeptidyl peptidase 4 inhibitors).mp. [mp=title, book title, abstract, original title, name of substance word, subject heading word, floating sub-heading word, keyword heading word, organism supplementary concept word, protocol supplementary concept word, rare disease supplementary concept word, unique identifier, synonyms, population supplementary concept word, anatomy supplementary concept word] |
| 5 | exp *Dipeptidyl-Peptidase IV Inhibitors/ |
| 6 | 4 or 5 |
| 7 | 3 and 6 |

Clinical trials

AREA[ConditionSearch](Type 1 Diabetes) AND AREA[InterventionSearch](DPP-4 inhibitors)

Table 1: characteristics of excluded studies

| **Study** | **Reason for exclusion** | **Reference** |
| --- | --- | --- |
| ?, 2009 | not a trial | [PMID: 19739530](https://pubmed.ncbi.nlm.nih.gov/19739530/) |
| Bellin et al,. 2017 | population not type 1 diabetes | DOI: [10.1111/ajt.13979](https://doi.org/10.1111/ajt.13979) |
| Blaslov et al,. 2015 | not a trial | DOI: [10.1016/j.jdiacomp.2014.12.019](https://doi.org/10.1016/j.jdiacomp.2014.12.019) |
| Bolla et al,. 2020 | intervention not DPP-4 inhibitors over insulin | DOI: [10.1210/clinem/dgaa791](https://doi.org/10.1210/clinem/dgaa791) |
| Bruzetti et al,. 2015 | not a trial | DOI: [10.1002/dmrr.2717](https://doi.org/10.1002/dmrr.2717) |
| schopman et al,. 2015 | duration less than 12 weeks | DOI:10.1111/dom.12453 |
| Curovic et al,. 2023 | duration less than 12 weeks | DOI: [10.2337/dc22-1699](https://doi.org/10.2337/dc22-1699) |
| Dib et al,. 2011 | unfinished trial | CTG: [NCT01559025](https://clinicaltrials.gov/study/NCT01559025) |
| Donath et al., 2014 | intervention not DPP-4 inhibitors over insulin | CTG:  [NCT02127047](https://clinicaltrials.gov/show/NCT02127047) |
| Ellis et al,. 2011 | duration less than 12 weeks | DOI: [10.1111/j.1464-5491.2011.03331.x](https://doi.org/10.1111/j.1464-5491.2011.03331.x) |
| Farngren et al., 2012 | duration less than 12 weeks | DOI: [10.1210/jc.2012-2332](https://doi.org/10.1210/jc.2012-2332) |
| Fengyi et al,. 2015 | text not in English | [ChiCTR-IPR-15005986](https://www.chictr.org.cn/showproj.html?proj=10439) |
| Foley et al,. 2008 | duration less than 12 weeks | DOI: [10.1055/s-2008-1078754](https://doi.org/10.1055/s-2008-1078754) |
| Garg et al,. 2013 | Duplicate | DOI: [10.1089/dia.2014.1514](https://doi.org/10.1089/dia.2014.1514) |
| Giampietro et al,. 2013 | intervention not DPP-4 inhibitors over insulin | DOI: [10.2147/DDDT.S38346](https://doi.org/10.2147/dddt.s38346) |
| griffin et al,. 2014 | Duplicate | DOI: [10.1016/S2213-8587(14)70115-9](https://doi.org/%2010.1016/S2213-8587(14)70115-9) |
| grill et al,. 2021 | Duplicate | DOI: [10.1210/clinem/dgab411](https://doi.org/10.1210/clinem/dgab411) |
| Grill et al,. 2021 | Duplicate | DOI: [10.1210/clinem/dgab411](https://doi.org/10.1210/clinem/dgab411) |
| Haidar et al,. 2023 | intervention not DPP-4 inhibitors over insulin | DOI: [10.2337/dc22-2297](https://doi.org/10.2337/dc22-2297) |
| Hals et al,. 2019 | Comparator not on top of insulin | DOI: [10.1111/dom.13797](https://doi.org/10.1111/dom.13797) |
| hari kumar et al,. 2013 | Duplicate | DOI: [10.1016/j.diabres.2013.01.020](https://doi.org/10.1016/j.diabres.2013.01.020) |
| Johansen et al,. 2014 | population not type 1 diabetes | DOI: [10.2337/dc13-1523](https://doi.org/10.2337/dc13-1523) |
| Laursen et al,. 2023 | duration less than 12 weeks | DOI: [10.1111/dom.15180](https://doi.org/10.1111/dom.15180) |
| liang et al,. 2021 | Duplicate | DOI: [10.1210/clinem/dgab413](https://doi.org/10.1210/clinem/dgab413) |
| Luo et al,. 2015 | text not in English | DOI: [10.11817/j.issn.1672-7347.2015.10.006](https://doi.org/10.11817/j.issn.1672-7347.2015.10.006) |
| Lynch et al,. 2021 | population not type 1 diabetes | DOI: [10.1159/000502130](https://doi.org/10.1159/000502130) |
| Pieber et al,. 2013 | duration less than 12 weeks | CTG: [NCT01782261](https://clinicaltrials.gov/study/NCT01782261?tab=table) |
| Pinheiro et al,. 2023 | not a trial | DOI: [10.20945/2359-3997000000652](https://doi.org/10.20945/2359-3997000000652) |
| Rabinovitch et al,. 2023 | not a trial | DOI: [10.3389/fendo.2023.1171886](https://doi.org/10.3389/fendo.2023.1171886) |
| Sadner, 2009 | population not type 1 diabetes | ISSN [09350020](https://www-scopus-com/record/display.uri?eid=2-s2.0-77950209175&origin=resultslist&sort=plf-f&src=s&sid=3acf58a30350c705f9ac6c154f9dfb61&sot=a&sdt=a&s=SOURCE-ID+%2821100855845%29&sl=23&sessionSearchId=3acf58a30350c705f9ac6c154f9dfb61&relpos=636) |
| Sudan et al,. 2021 | population not type 1 diabetes | DOI: [10.1016/j.dsx.2021.102197](https://doi.org/10.1016/j.dsx.2021.102197) |
| Su et al,. 2014 | population not type 1 diabetes | DOI: [10.3892/etm.2014.1545](https://doi.org/10.3892/etm.2014.1545) |
| Underland et al., 2017 | duration less than 12 weeks | DOI: [10.1177/1932296817699847](https://doi.org/10.1177/1932296817699847) |
| yanai et al,. 2018 | not a trial | DOI: [10.1016/j.diabet.2017.05.002](https://doi.org/10.1016/j.diabet.2017.05.002) |
| Yan et al,. 2023 | Comparator not on top of insulin | DOI: [10.1038/s41392-023-01369-9](https://doi.org/10.1038/s41392-023-01369-9) |
| Yang et al,. 2014 | unfinished trial | CTG: [NCT02307695](https://clinicaltrials.gov/show/NCT02307695) |
| Zhang et al,. 2020 | Comparator not on top of insulin | DOI: [10.1002/dmrr.3298](https://doi.org/10.1002/dmrr.3298) |
| Zhou 2019 | population not type 1 diabetes | DOI: [10.1530/EC-18-0523](https://doi.org/10.1530/ec-18-0523) |

###### Figure 1: Forest plot for subgroup analysis concerning treatment duration for HbA1c in the DPP-4 inhibitors vs. placebo group


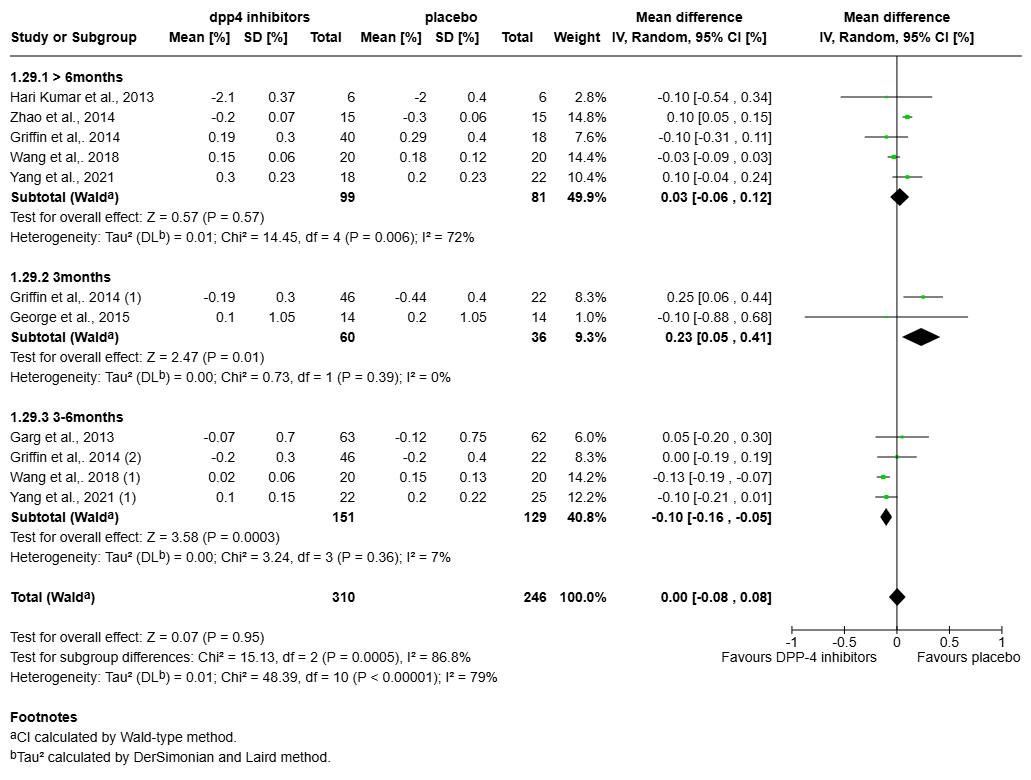


###### Figure 2: Forest plot for subgroup analysis concerning BMI for HbA1c in the DPP-4 inhibitors vs. placebo group


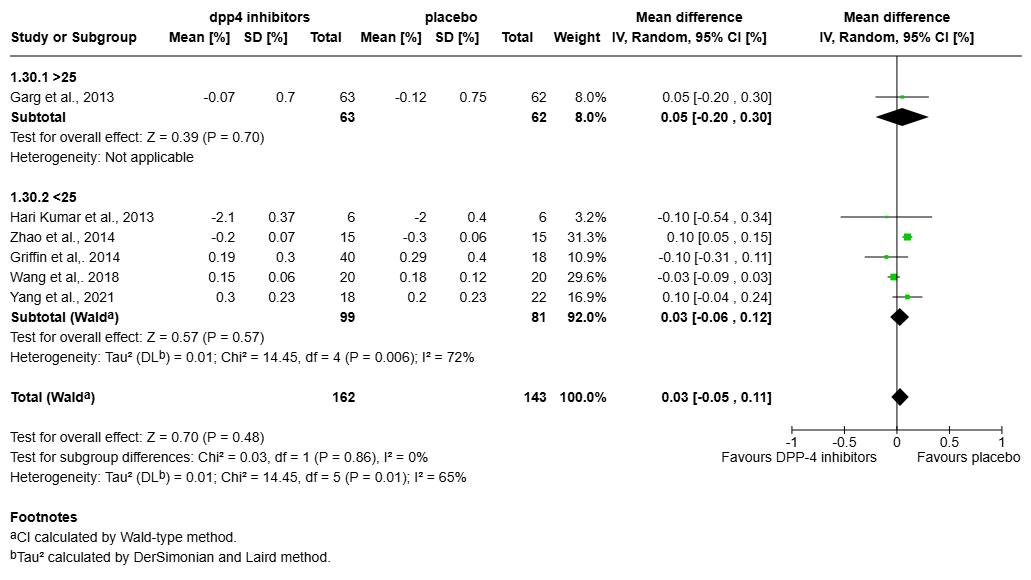


###### Figure 3: Forest plot for subgroup analysis concerning diabetes duration for HbA1c in the DPP-4 inhibitors vs. placebo group


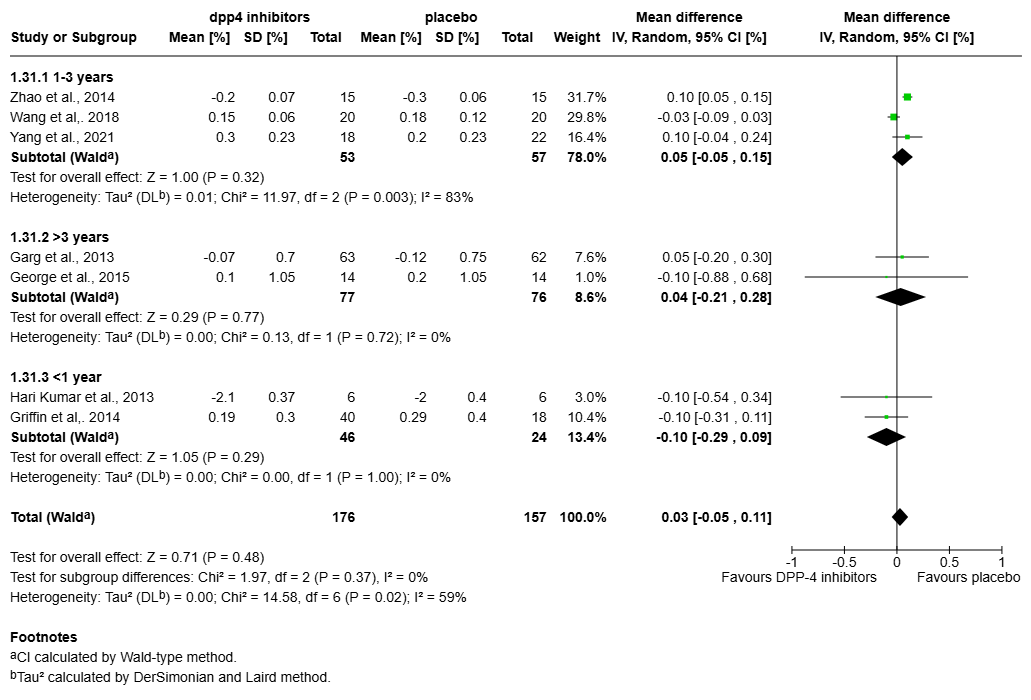


###### Figure 4: Forest plot for HbA1c in the Sitagliptin +/- Lansoprazole vs. placebo group


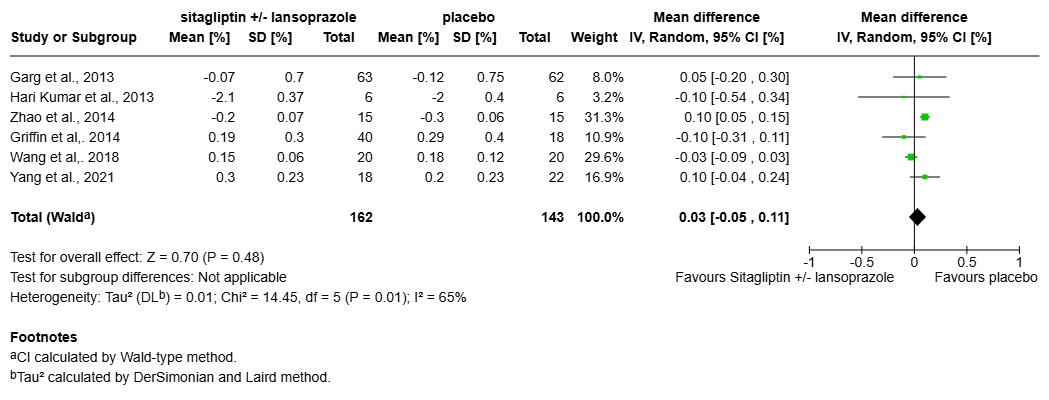


###### Figure 5: Forest plot for subgroup analysis concerning treatment duration for HbA1c in the Sitagliptin +/- Lansoprazole vs. placebo group


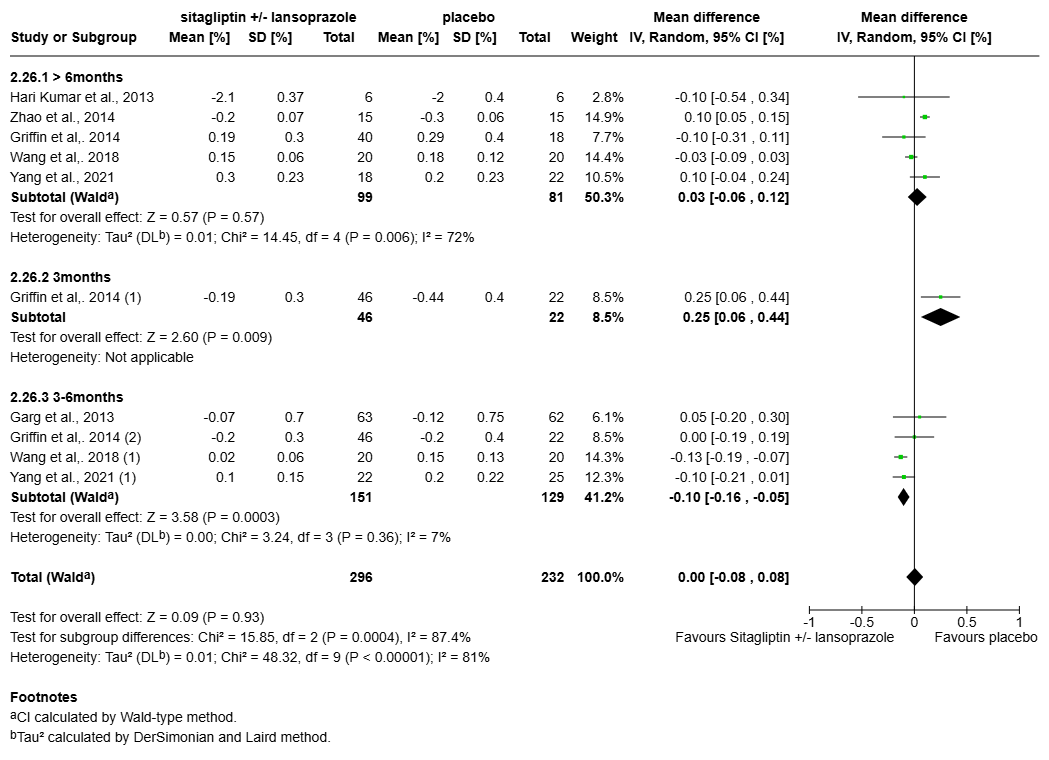


###### Figure 6: Forest plot for subgroup analysis concerning BMI for HbA1c in the Sitagliptin +/- Lansoprazole vs. placebo group


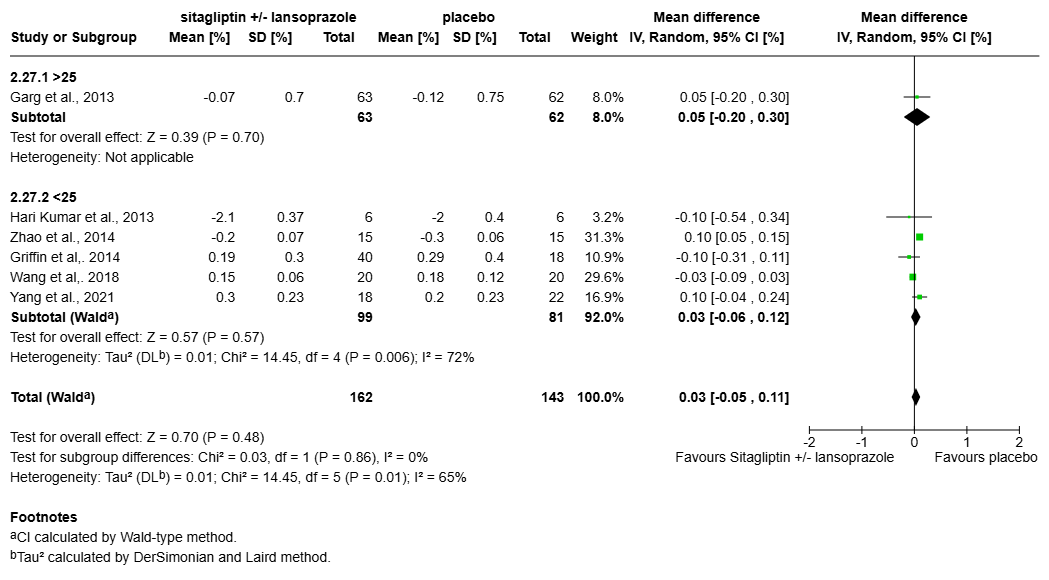


###### Figure 7: Forest plot for subgroup analysis concerning diabetes onset for HbA1c in the Sitagliptin +/- Lansoprazole vs. placebo group
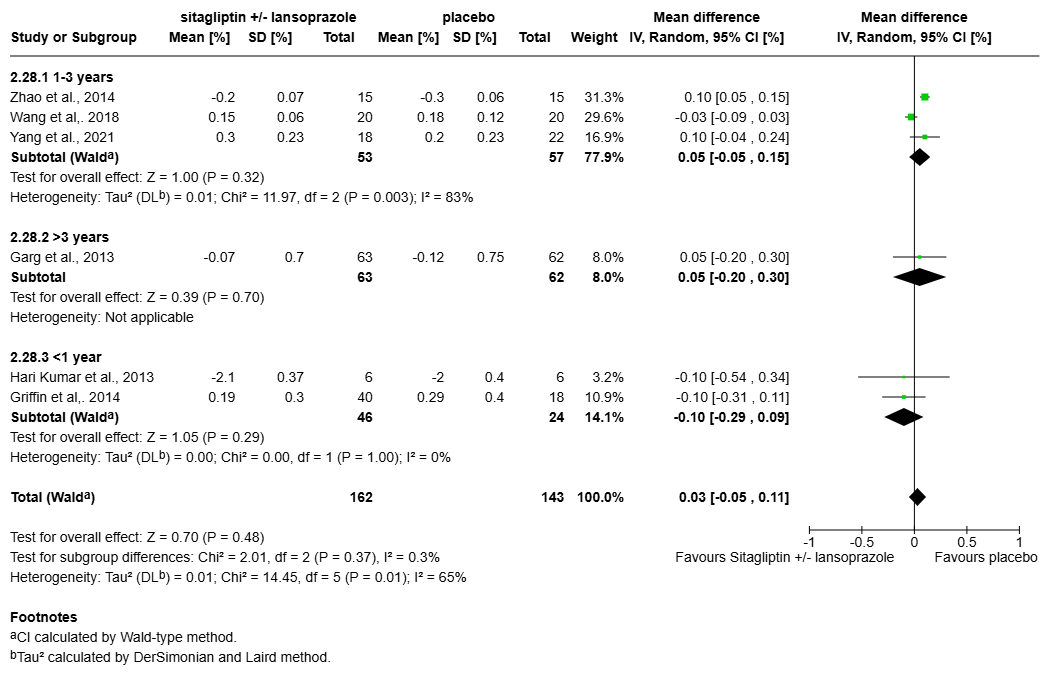


###### Figure 8: Forest plot for HbA1c in the Sitagliptin vs. placebo group
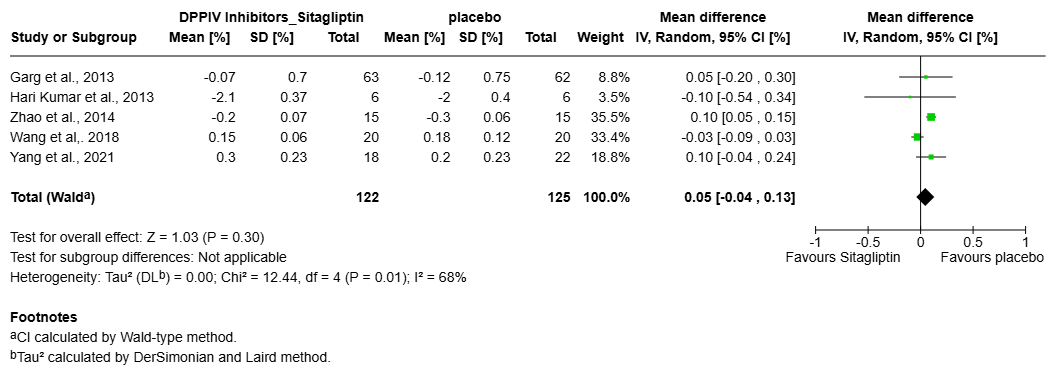


###### Figure 9: Forest plot for subgroup analysis concerning treatment duration for HbA1c in the Sitagliptin vs. placebo group
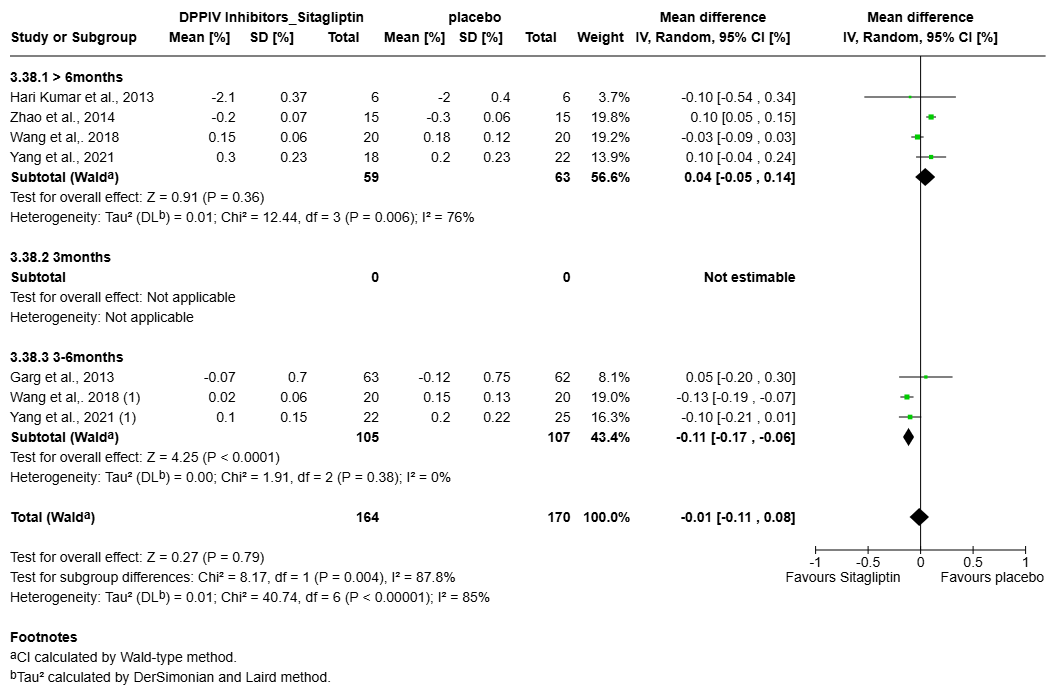


###### Figure 10: Forest plot for subgroup analysis concerning BMI for HbA1c in the Sitagliptin vs. placebo group
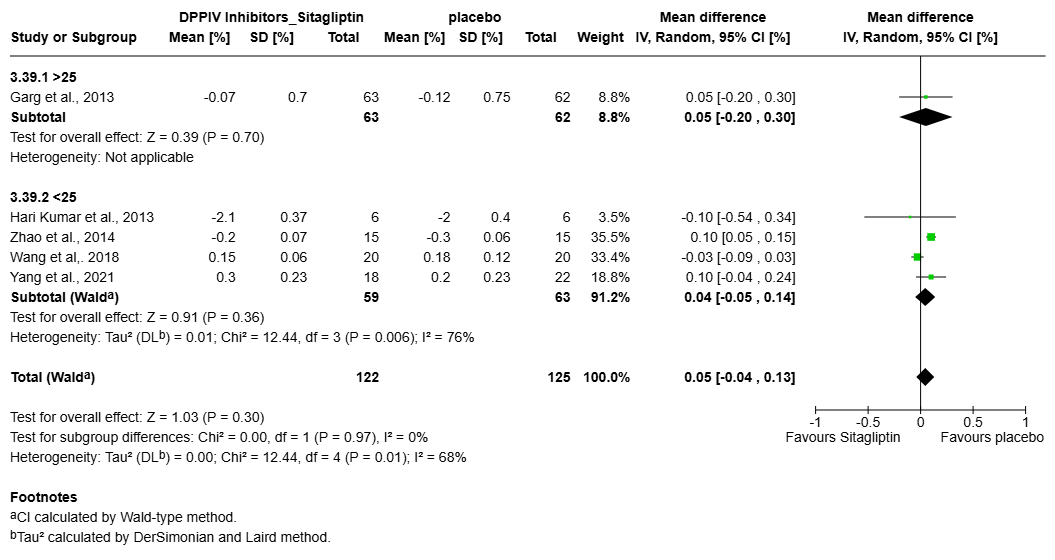


###### Figure 11: Forest plot for subgroup analysis concerning diabetes onset for HbA1c in the Sitagliptin vs. placebo group
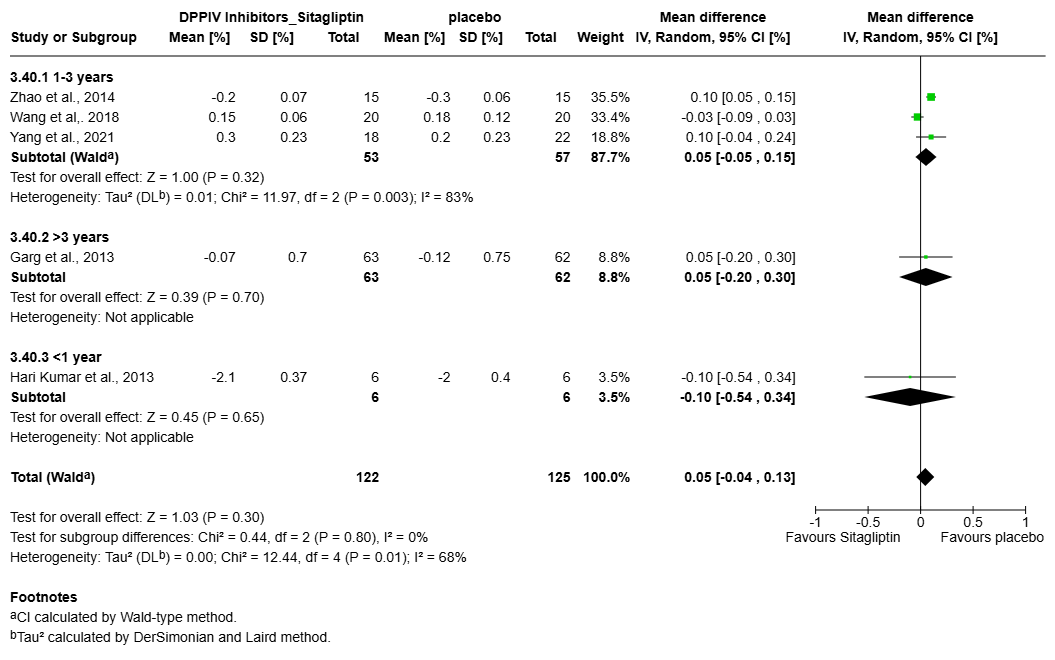


###### Figure 12: Forest plot for fasting C peptide in the Dpp-4 inhibitors vs. placebo group
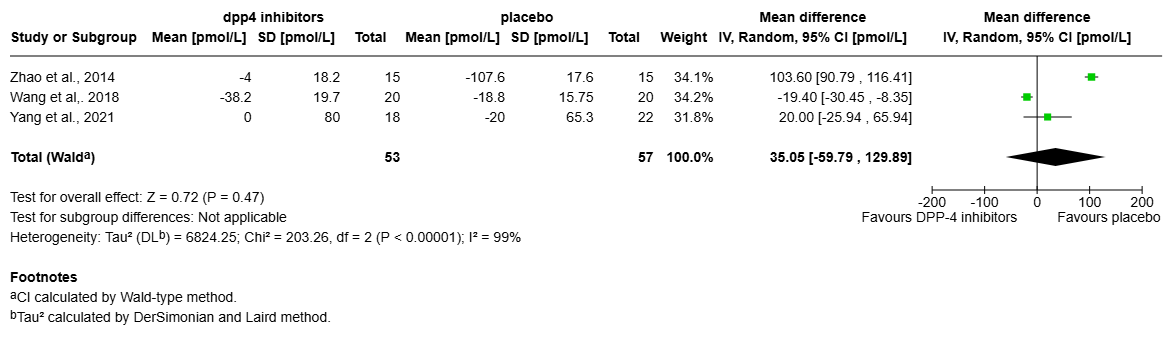


###### Figure 13: Forest plot for subgroup analysis concerning treatment duration for fasting C peptide in the DPP-4 inhibitors vs. placebo group
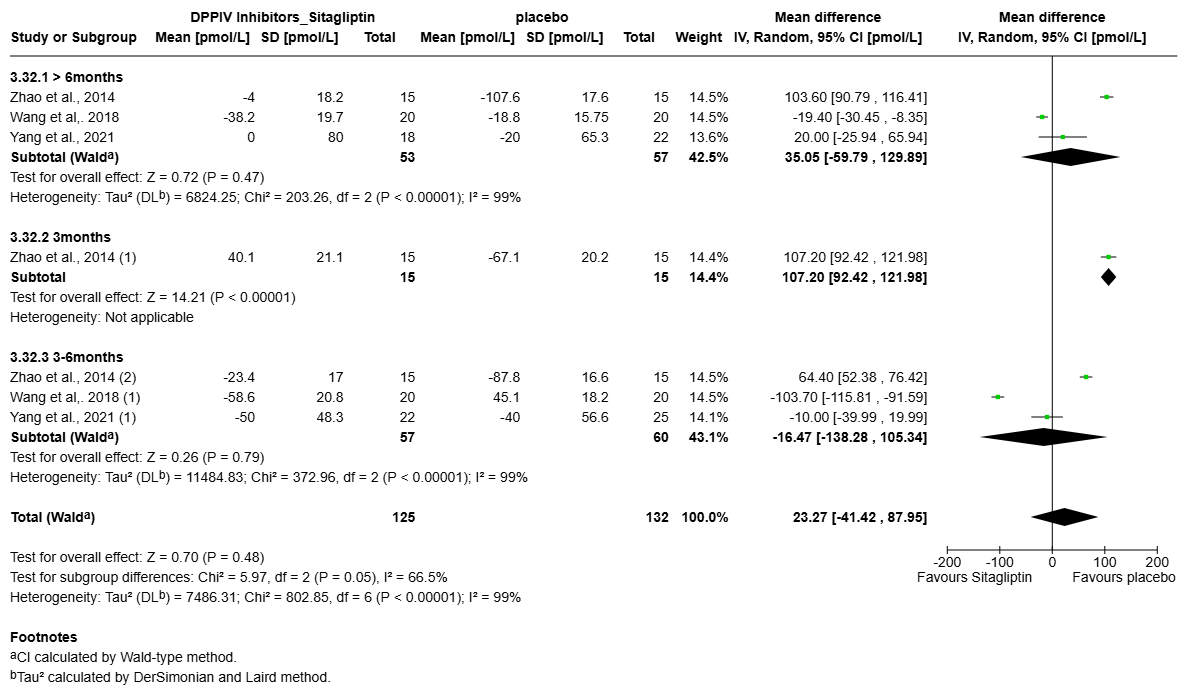


###### Figure 14: Forest plot for post prandial C-peptide in the DPP-4 inhibitors vs. placebo group
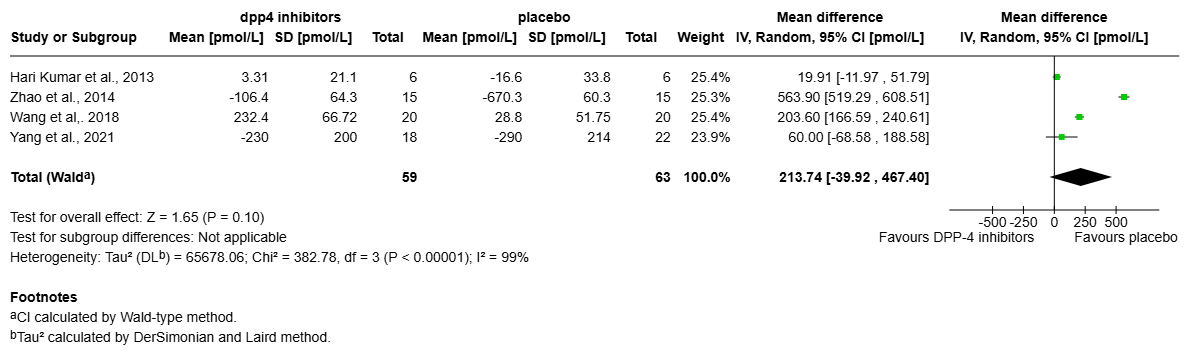


###### Figure 15: Forest plot for subgroup analysis concerning treatment duration for post prandial C-peptide in the DPP-4 inhibitors vs. placebo group
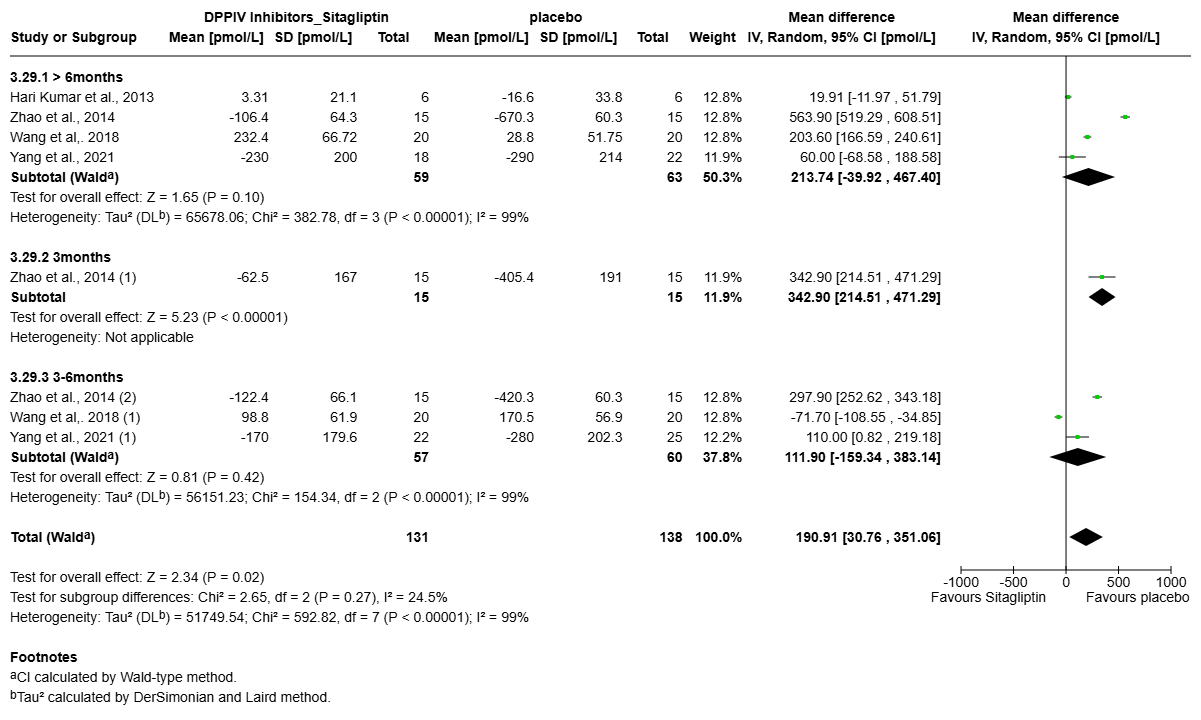


###### Figure 16: Forest plot for subgroup analysis concerning diabetes onset for post prandial C-peptide in the DPP-4 inhibitors vs. placebo group
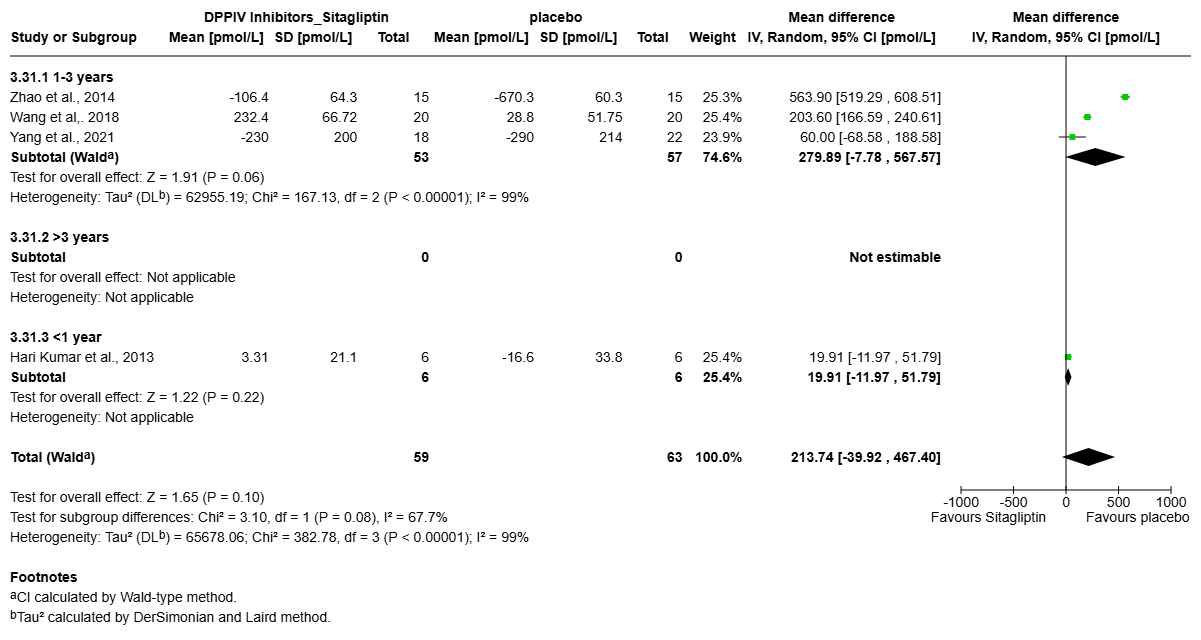


###### Figure 17: Forest plot for ΔC-peptide in the DPP-4 inhibitors vs. placebo group
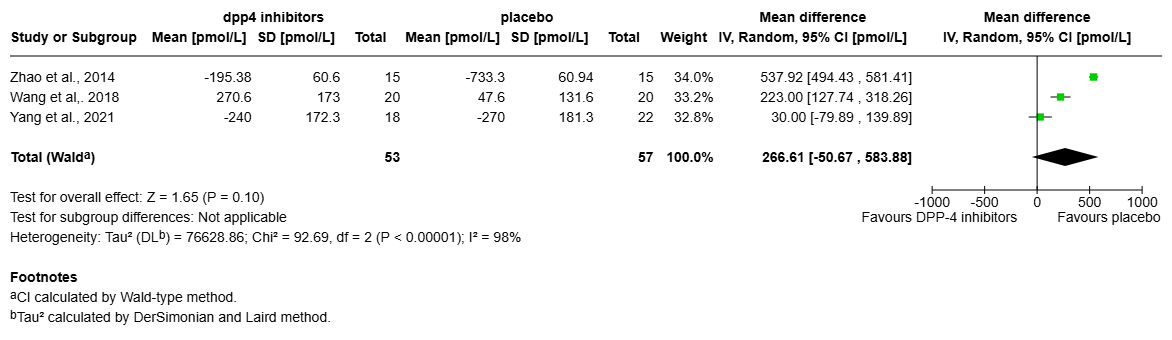


###### Figure 18: Forest plot for subgroup analysis concerning treatment duration for ΔC-peptide in the DPP-4 inhibitors vs. placebo group
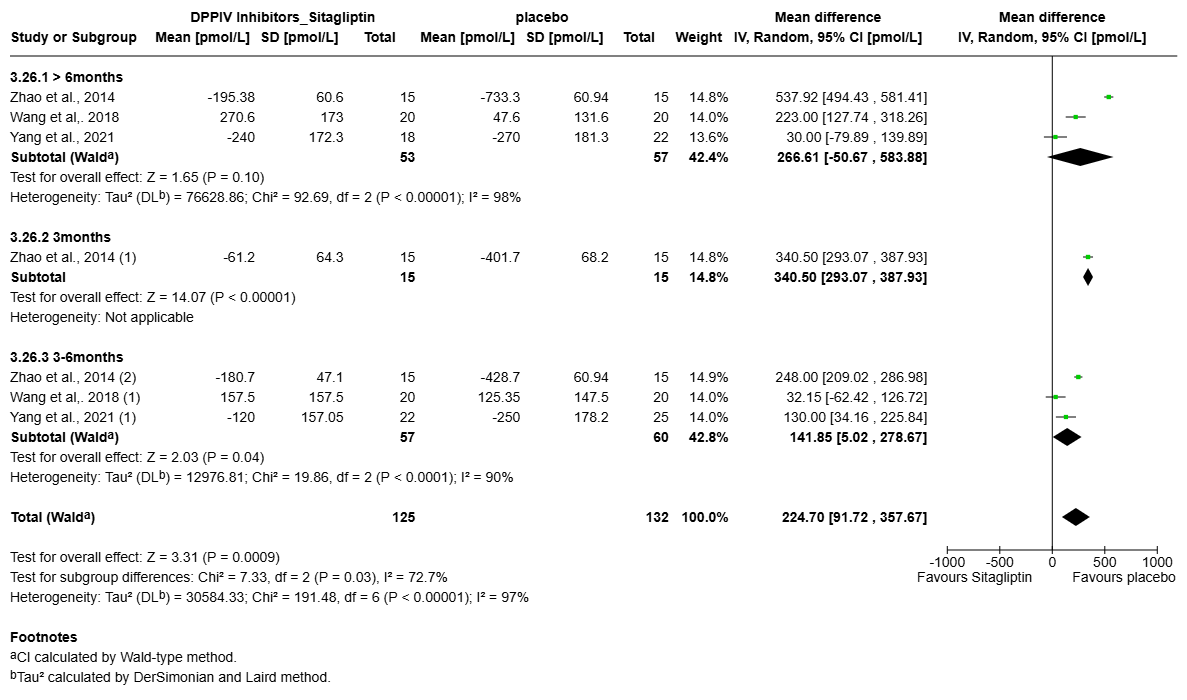


###### Figure 19: Forest plot for C peptide AUC 2 hours post prandial in the Dpp-4 inhibitors vs. placebo group
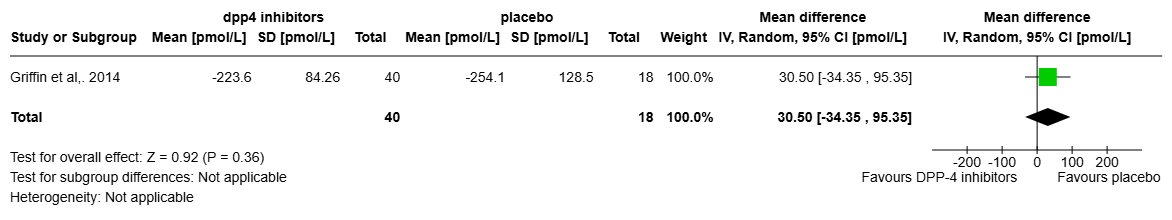


###### Figure 20: Forest plot for C peptide AUC 2 hours post prandial in the Dpp-4 inhibitors vs. placebo group
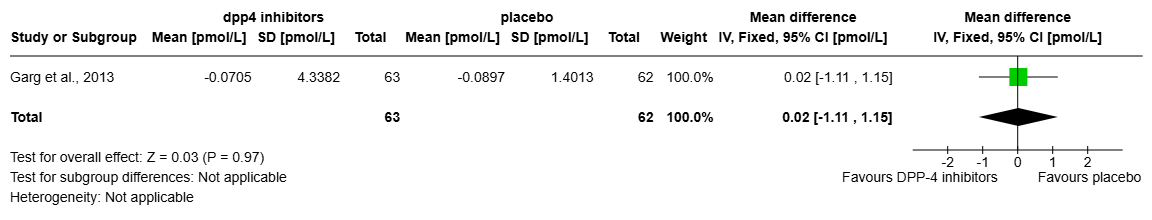


###### Figure 21: Forest plot for subgroup analysis concerning treatment duration for insulin units/day in the DPP-4 inhibitors vs. placebo group
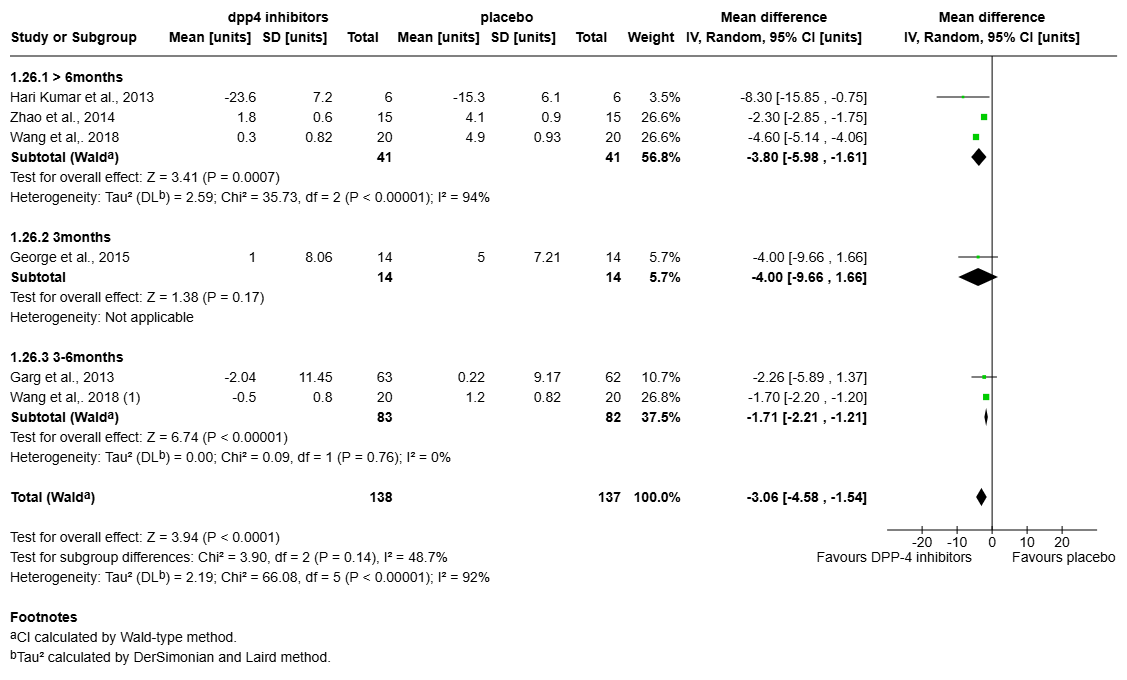


###### Figure 22: Forest plot for subgroup analysis concerning BMI for insulin units/day in the DPP-4 inhibitors vs. placebo group
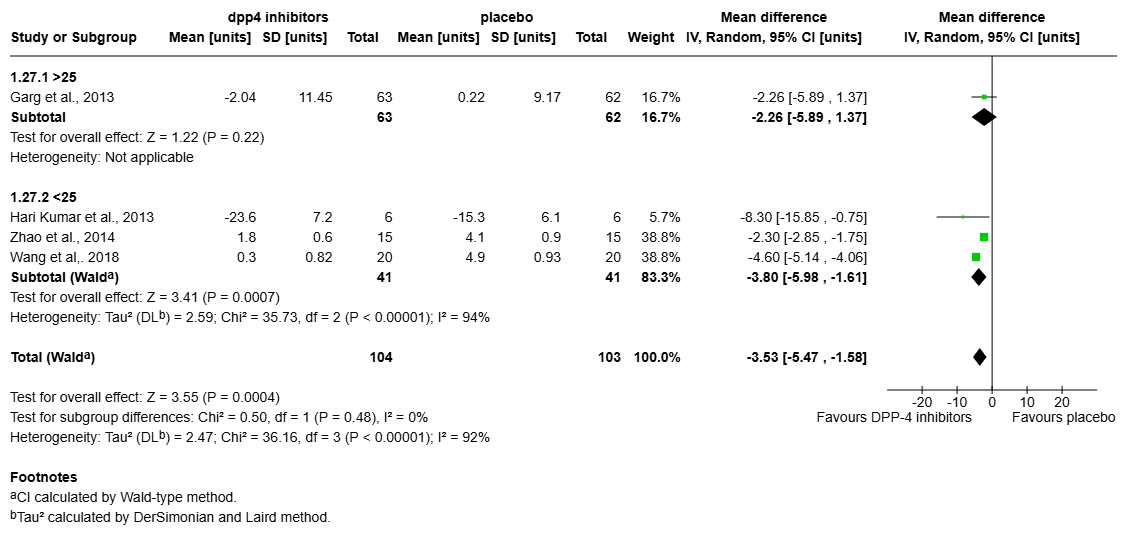


###### Figure 23: Forest plot for subgroup analysis concerning diabetes duration for insulin units/day in the DPP-4 inhibitors vs. placebo group
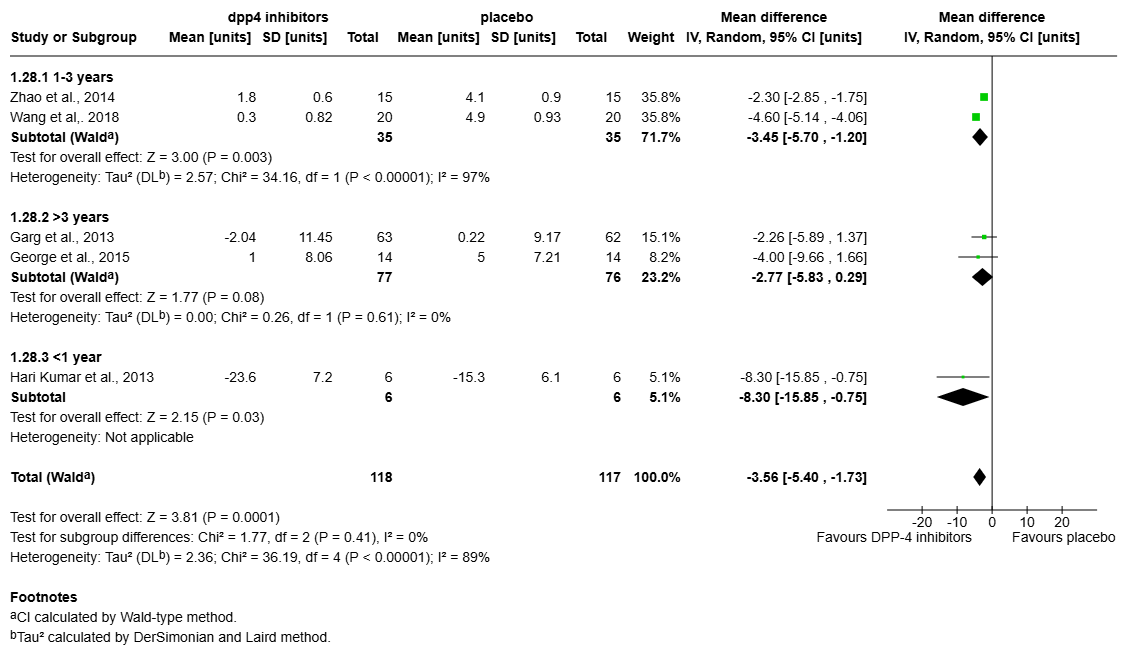


###### Figure 24: Forest plot for insulin units/day in the Sitagliptin vs. placebo group
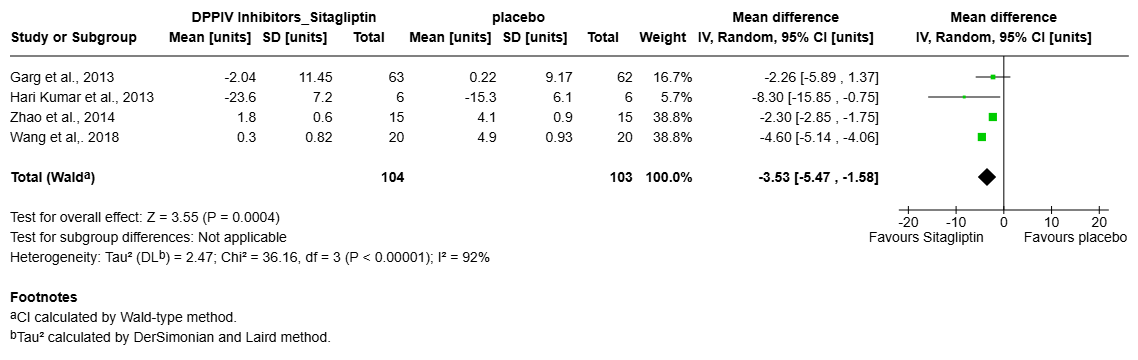


###### Figure 25: Forest plot for subgroup analysis concerning treatment duration for insulin units/day in the Sitagliptin vs. placebo group
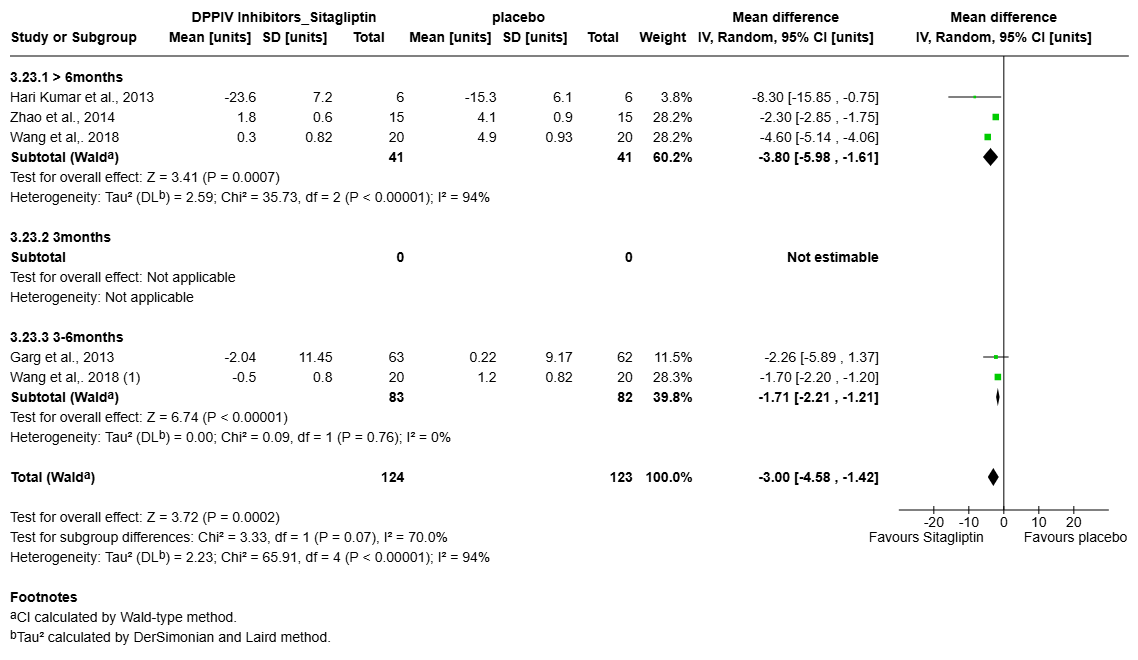


###### Figure 26: Forest plot for subgroup analysis concerning BMI for insulin units/day in the Sitagliptin vs. placebo group
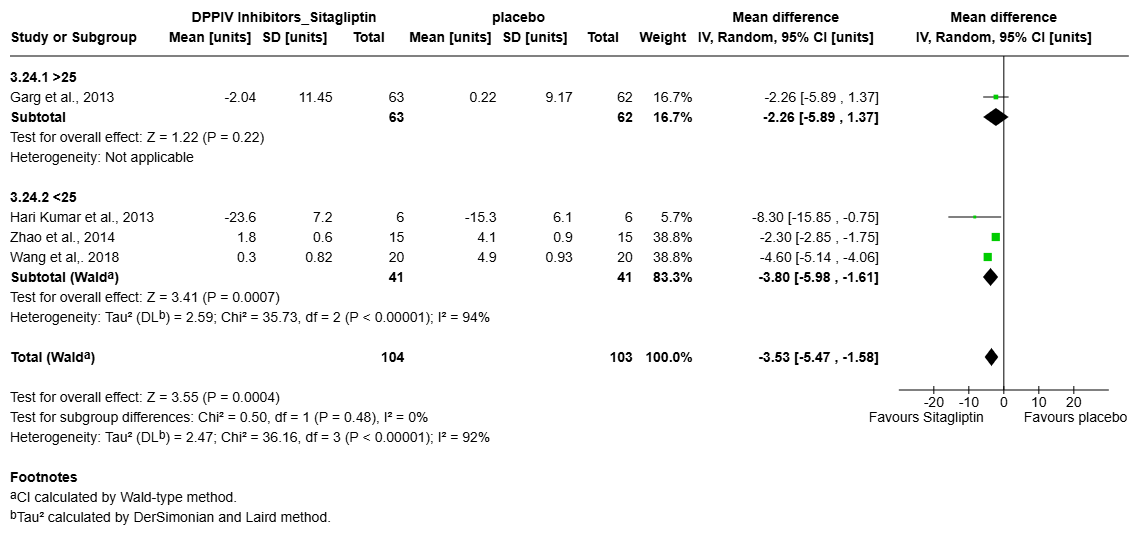


###### Figure 27: Forest plot for subgroup analysis concerning diabetes duration for insulin units/day in the Sitagliptin vs. placebo group
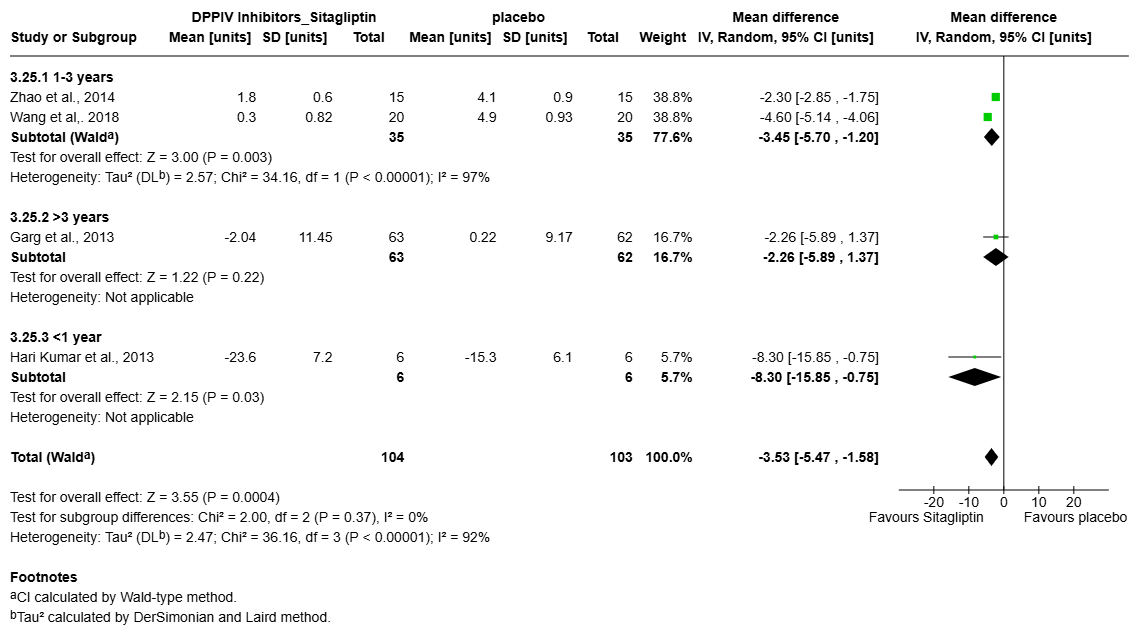


###### Figure 28: Forest plot for basal insulin units/day in the DPP-4 inhibitors vs. placebo group
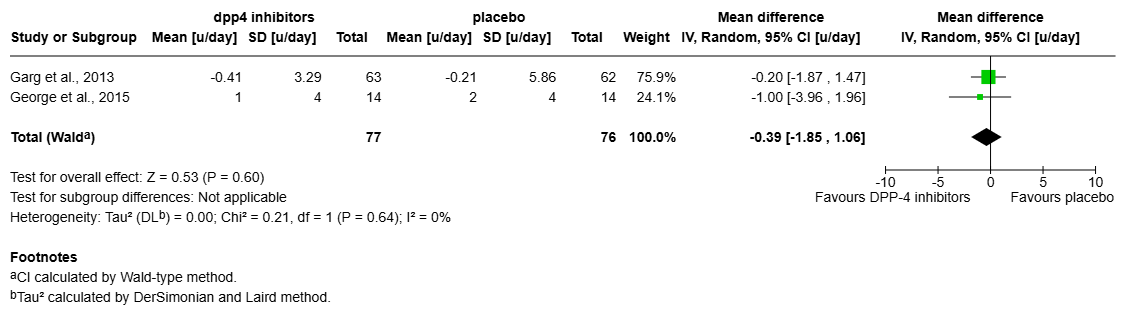


###### Figure 29: Forest plot for bolus insulin units/day in the DPP-4 inhibitors vs. placebo group
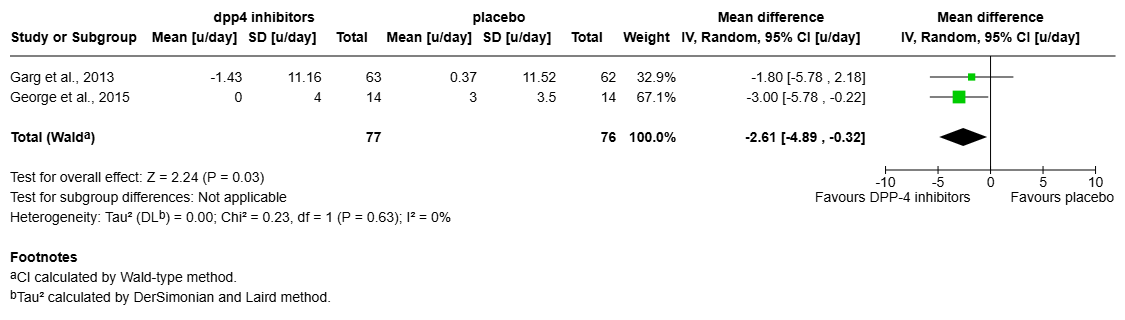


###### Figure 30: Forest plot for insulin units adjusted for weight in the DPP-4 inhibitors vs. placebo group


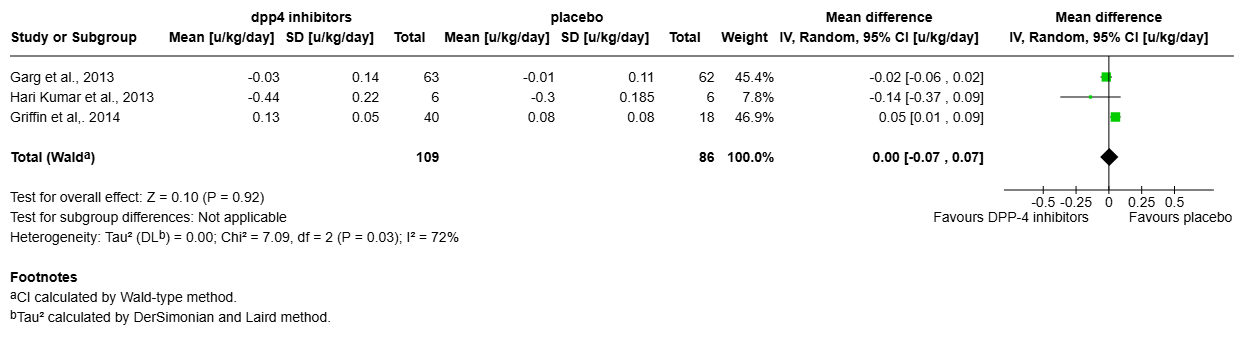

Supplement: S2 Appendix — (DOCX) [file pone.0332191.s002.docx]
